# Integrating RNA-seq and scRNA-seq to explore the biological significance of NAD + metabolism-related genes in the initial diagnosis and relapse of childhood B-cell acute lymphoblastic leukemia

**DOI:** 10.3389/fimmu.2022.1043111

**Published:** 2022-11-11

**Authors:** Chao Lin, Jia-Qi Xu, Gui-Chao Zhong, Hui Chen, Hong-Man Xue, Mo Yang, Chun Chen

**Affiliations:** ^1^ Department of Pediatrics, The Seventh Affiliated Hospital, Sun Yat-Sen University, Shenzhen, China; ^2^ Department of Nephrology, Center of Kidney and Urology, The Seventh Affiliated Hospital, Sun Yat-Sen University, Shenzhen, China; ^3^ Department of Pediatrics, Shenzhen People’s Hospital (The Second Clinical Medical College, Jinan University, The First Affiliated Hospital, Southern University of Science and Technology), Shenzhen, China; ^4^ Scientific Research Center, The Seventh Affiliated Hospital, Sun Yat-Sen University, Shenzhen, China

**Keywords:** B-cell acute lymphoblastic leukemia, NAD metabolism-related genes, biomarkers, single cell RNA sequencing, cell cluster

## Abstract

**Background:**

Nicotinamide Adenine Dinucleotide (NAD) depletion is reported to be a potential treatment for B-cell Acute Lymphoblastic Leukemia (B-ALL), but the mechanism of NAD metabolism-related genes (NMRGs) in B-ALL relapse remains unclear.

**Methods:**

Transcriptome data (GSE3912), and single-cell sequencing data (GSE130116) of B-ALL patients were downloaded from Gene Expression Omnibus (GEO) database. NMRGs were sourced from Kyoto Encyclopedia of Genes and Genomes (KEGG) and Reactome databases. Further, the differentially expressed NMRGs (DE-NMRGs) were selected from the analysis between initial diagnosis and relapse B-ALL samples, which further performed functional enrichment analyses. The biomarkers were obtained through random forest (RF) algorithm and repeated cross validation. Additionally, cell type identification by estimating relative subsets of RNA transcripts (CIBERSORT) algorithm was used to evaluate the immune cell differences between the initial diagnosis and relapse samples, and the correlations between biomarkers and gene markers of differential immune cells were analyzed. Furthermore, single cell RNA sequencing was conducted in the GSE130116 dataset to find key cell clusters. In addition, according to biomarkers expressions, cell clusters were categorized into high and low biomarker expression groups, and Gene Set Enrichment Analysis (GSEA) analysis was performed on them. Finally, the cell clusters with the highest expression of biomarkers were selected to explore the roles of biomarkers in different cell clusters and identify transcription factors (TFs) influencing biological markers.

**Results:**

23 DE-NMRGs were screened out, which were mainly enriched in nucleoside phosphate metabolic process, nucleotide metabolic process, and Nicotinate and nicotinamide metabolism. Moreover, 3 biomarkers (NADSYN1, SIRT3, and PARP6) were identified from the machine learning. CIBERSORT results demonstrated that four types of immune cells (B Cells naive, Monocyte, Neutrophils, and T cells CD4 memory Activated) were significantly different between the initial diagnosis and the relapse B-ALL samples, and there were strong correlations between biomarkers and differential immune cells such as positive correlation between NADSYN1 and B Cells naive. The single cell analyses showed that the biomarkers were highly expressed in common myeloid progenitors (CMP), granulocyte-macrophage progenitor (GMP), and megakaryocyte-erythroid progenitor (MEP) cell clusters. Gene set enrichment analysis (GSEA) results indicated that 55 GO terms and 3 KEGG pathways were enriched by the genes in high and low biomarker expression groups. It was found that TF CREB3L2(+) was significantly reduced in the high expression group, which may be the TF affecting biomarkers in the high expression group.

**Conclusion:**

This study identified NADSYN1, SIRT3, and PARP6 as the biomarkers of B-ALL, explored biological significance of NMRGs in the initial diagnosis and relapse of B-ALL, and revealed mechanism of biomarkers at the level of the single cell.

## Introduction

Acute lymphoblastic leukemia (ALL) relates to the situation that immature lymphoid progenitor cells are expanded clonally and lymphocyte sources become abnormal (1). The American Cancer Society (ACS) reported 5,290 new adult and pediatric cases of ALL that resulted in 1,580 deaths in 2021 (2). Despite the obviously declined ALL incidence with time, ALL remains the primary tumor type for children (3). ALL is composed of the B-cell and T-cell lineages, with the former taking up about 85% of pediatric ALL (4). Chemotherapy and the Hematopoietic Stem Cell Transplantation (HSCT) technique have developed rapidly, as a result, pediatric ALL presents the cure rate of nearly 90% (5). Its overall survival has progressed remarkably in the long term, while 15–20% of patients still suffer ALL relapse, that largely explains the increase in mortality for ALL patients (6). Therefore, the identification of potential biomarkers of relapse in B-ALL patients is crucial to improve the prognosis.

Nicotinamide adenine dinucleotide (NAD +) acts as a pivotal coenzyme in the redox reaction, and also crucially constitutes the energy metabolism. In traditional concept, nicotinamide nucleotide metabolism is very static, which mainly puts emphasis on the way different forms of NAD (oxidized or reduced) interconvert with the nicotinamide adenine dinucleotide phosphate(NADP) (7, 8). Nevertheless, according to researches in the past thirty years, NAD possesses complex and dynamic biological progresses, such as metabolism, transport, and function. NAD is capable of being converted into NADP, nicotinic acid adenine dinucleotide phosphate (NAADP), and cyclic ADP-ribose (cADPR), thereby remarkably impacting the energy transduction and the cell signaling. Besides, the degradation products of NAD, like nicotinamide and positive methyl nicotinamide, are also pivotal modulators of the epigenetics, energy metabolism, as well as disease states (9, 10). The pathway metabolites NAD can be the substrates for various enzymes like PARPs, which can impact the cellular homeostasis from various aspects (11, 12). What’s more, KPT-9274 acts as a different nicotinamide phosphoribosyl transferase (NAMPT) inhibitor, and NAMPT limits the NAD rescue biosynthesis rate, and crucially affects the energy metabolism. KPT-9274 could inhibit NAMPT for NAD + depletion, thereby inhibiting B-ALL cell growth (13). Despite this, researches fail to well explain the impact exerted by NAD + metabolism-related genes on the recurrence of B-ALL.

Taken together, this study explored the biological significance of NAD + metabolism in the initial diagnosis and recurrence of B-ALL based on the differential NAD + metabolism-related genes in the initial diagnosis and recurrence of B-ALL patients, searched potential biomarkers of B-ALL, revealed the molecular mechanism at the single-cell level, and deepened the understanding of the pathogenesis of B-ALL.

## Materials and methods

### Data sources

The workflow chart of this study was shown in **Supplementary Figure S1**. Transcriptome data of B-ALL patients were selected from GSE3912, which contained Fragments Per Kilobase of exon model per Million mapped fragments (FPKM) gene-expression matrix of bone marrow samples in 32 first diagnosis children patients and 54 relapses samples, and was sourced *via* GEO database. GSE130116, single-cell RNA-sequencing libraries of bone marrow from 7 pairs of pediatric B-ALL patients with initial diagnosis or relapse with gene expression matrix as FPKM value, was also acquired from GEO database.

In addition, according to the research of Li et al., NAD+ metabolization-related genes (NMRGs) were selected from KEGG database (Pathway: hsa00760) and Reactome Database (R-HSA-196807) (14). After combination and duplication eradication, a total number of 51 NMRGs were obtained. Moreover, complete clinical and expression information of 9 pediatric B-ALL patients was acquired from the TARGET database. Clinical and Demographic characteristics of B-ALL patients from GSE3912 dataset, GSE130116 dataset, and TARGET database were exhibited in **Supplementary Table S1**.

### Screening of differentially expressed NMRGs

Based on 51 obtained NMRGs, the expression levels of NMRGs were found in the GSE3912 dataset, and the differentially expressed NMRGs (DE-NMRGs) between the initial diagnosis and relapse samples were compared using R-package limma (Version 3.48.3), with |log_2_FC| > 0.5, p Value < 0.05 as screening criteria (15). Next, the screened DE-NMRGs were performed functional enrichment analyses by ClusterProfiler (version 4.0.2). The enriched results satisfied p < 0.05 and count ≥ 1 were regarded as significantly enriched.

### Identification of biomarkers and correlation with clinical characteristics

R package Boruta (version 7.0.0) was firstly used to determine the importance of each DE-NMRGs through random forest (RF) algorithm and select DE-NMRGs with the confirmed importance (16). Then, the repeated cross validation was applied to the selected DE-NMRGs to further screen the most important genes which were considered as biomarkers. In order to investigate the ability of biomarkers to extinguish initial diagnosis from relapse samples, logistic regression fitting was employed on the biomarkers, and receiver operating characteristic (ROC) curves for logistic regression model was plotted using R package pROC (Version 1.18.0) (17). The ggplot2 (version 3.3.5) was employed to visualize the expressions of biomarkers in initial diagnosis and relapse samples (18).

Furthermore, to investigate the difference in biomarker expressions between different clinicopathological characteristics of B-ALL patients, biomarkers expression information and clinical characteristics (CNS Status, Ethnicity, Gender, Race) of the 9 samples from TARGET database were extracted and compared the expression levels of biomarkers in different clinical traits by ggplot2 (Version 3.3.5).

### Tumor microenvironment analysis

TME cells were important components in tumor tissues, and an increasing number of evidence had proven their clinicopathological significance in prognosis and treatment of cancers. Cell type identification by estimating relative subsets of RNA transcripts (CIBERSORT) was first employed to compute the proportions of 22 types of immune cells in the samples from initially diagnosed and relapse groups, and the results were visualized by tidyverse (version 1.3.1) (19). Then, the percentage of each immune cell was compared between the first diagnosis and relapse group and plotted by vioplot (version 0.3.7) (https://github.com/TomKellyGenetics/vioplot) to distinguish the differentially expressed immune cells (DEIs) (20). Furthermore, corrplot (version 0.92) (https://github.com/taiyun/corrplot) was applied to detect the correlations between the 22 types of immune cells and the biomarkers, to find the immune cells significantly correlated with biomarkers (p < 0.05) (21).

In addition, the overlap analysis was conducted between the DEIs and the immune cells correlated with biomarkers, and the intersection was regarded as key DEIs. Subsequently, based on the expression data of the biomarkers, the correlations between biomarkers and corresponding gene markers of key DEIs were determined by Pearson correlation analysis.

### Cluster and pseudotime analysis

Seurat (version 4.1) in R package was employed to conduct scRNA-seq quality control on the 7 pairs of B-ALL samples in GSE130116, with nFeature_RNA > 100, percent.mt < 5, and nCount_RNA > 3 as screening criteria (22). Then, the filtered genes were further conducted variance analysis, and the top 2,000 genes whose expression varied significantly among cells were identified and used for subsequent cell type identification. The principal components for highly variable genes were calculated. Based on the PCA result, principal components with p values less than 0.05 were used to identify clusters using the UMAP2 algorithm. Finally, ‘FindAllMarkers’ function was applied to search cluster markers with min.pct=0.2 and only.pos=TRUE. Cluster cell types were assigned according to cluster markers and cluster labels from the R package ‘SingleR’ (version 1.6.1) (23). In addition, the pseudotime analysis was employed by R package ‘monocle 2’ (version 2.20.0), and the cells were visualized in trajectory (24).

### Ligand-receptor analysis

The cell communication analysis was conducted through CellPhone DB database. Firstly, the numbers of ligand-receptor interaction and polymer between cell types were counted, and the interactions were subsequently filtered based on the thresholds of p value<=0.05 and log2 mean (Molecule 1, Molecule 2) >=0.1. Additionally, according to the filtered interaction pairs, the interactions analysis was employed on the cell types acquired from step 2.5.

### Confirmation of key cell clusters and gene set enrichment analysis

The average expressions of biomarkers in each cell cluster were calculated, and the expression levels of biomarkers in each cell cluster were ranked from high to low. The top 3 cell clusters with the highest biomarkers expressions were regarded as key cell clusters and performed consensus clustering based on the biomarkers to determine the optimal cluster number (K).

Moreover, the biomarker expression boxplots were drawn based on the clusters from the consensus cluster result to identify the high and low expression groups of biomarkers. The proportions of the high and low biomarker expression groups in the initial diagnosis and relapse groups were also analyzed. To further investigate the potential mechanism of biomarkers expression in the initial diagnosis and relapse groups, the GSEA was employed on the biomarkers in high and low expression groups by R clusterProfiler (version 3.18.1) (25). The enrichment files were c5.go.v7.4.entrez.gmt and c2.cp.kegg.v7.4.entrez.gmt, which were downloaded from http://www.gsea-msigdb.org/gsea/msigdb, and the selection thresholds were set as |NES|>1, NOM P<0.05, and q<0.25.

### Identification of potential transcription factors affecting the expressions of biomarkers

To explore TFs in high and low biomarker expression groups, the specific TFs of the expression groups were predicted by single-cell regulatory network inference and clustering (SCENIC), and the top 10 TFs of regulation specificity scores were illustrated by scatter plots and bar charts. In addition, the TFs in the top 10 TFs regulation specificity scores only existed in the high expression group were further conducted expression comparison between the expression groups to investigate the TFs that would affect the expressions of biomarkers.

### Validation of biomarkers expressions by quantitative real-time polymerase chain reaction

Sixteen bone marrow samples were divided into 2 groups (Initial diagnosis =10, and Relapse = 6). They were lysed by 1 mL of TRIzol Reagent (Life Technologies, CA, USA) respectively, and the total RNA was isolated following the manufacturer’s instructions. After detecting the concentration and the purity of RNA, qualified RNA was reverse-transcribed to cDNA using the SureScript-First-strand-cDNA-synthesis-kit (Genecopoeia, Guangzhou, China) prior to qRT-PCR. The qRT-PCR reaction consisted of 3 µl of cDNA, 5 µl of 2xUniversal Blue SYBR Green qPCR Master Mix (Servicebio, Wuhan, China), and 1 µl each of forward and reverse primer. PCR was performed in a BIO-RAD CFX96 Touch TM PCR detection system (Bio-Rad Laboratories, Inc., USA) under the thermal cycling conditions: 40 cycles at 95°C for 60 s, 95°C for 20 s, 55°C for 20 s, and 72°C for the 30s. The 2^-△△Ct^ method was used to calculate gene expressions, and Graphpad Prism 5 was applied to plot and calculated the statistic significance. Clinical characteristics of B-ALL patients from our study were exhibited in **Supplementary Table S2**. The primer sequences used in the current study were given in following **Supplementary Table S3**.

### Statistical analysis

All statistical analyses were conducted in R software (3.6.1) and Graphpad Prism 5. The data from different groups were compared by the Wilcoxon test and t-test. If not specifically stated p value < 0.05 was considered statistically significant.

## Results

### Identification and enrichment annotation of DE-NMRGs

**Figure 1:**
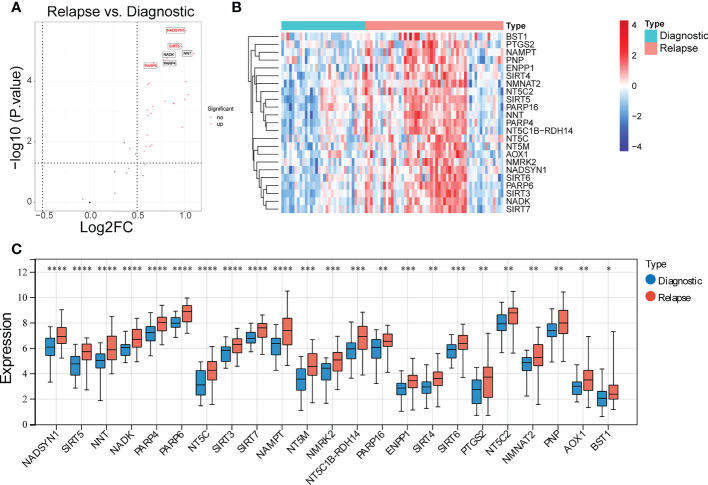
Identification of differentially expressed NAD+ metabolization-related genes (DE-NMRGs). **(A)** Volcano plot of DE-NMRGs between initially diagnosed and relapse samples, the red dots indicate up-regulated genes with the thresholds of |log2FC| > 0.5 and p < 0.05. **(B)** Heatmap of 23 DE-NMRGs in GSE3912. **(C)** The expression profile of 23 DE-NMRGs in GSE3912. * represents p < 0.05, ** represents p < 0.01, *** represents p< 0.001, **** represents p < 0.0001.

To identify the DE-NMRGs, expression levels of 33 NMRGs were extracted from GSE3912 according to the 51 NMRGs from previous analysis. The differential analysis showed that 23 DE-NMRGs were screened out between initially diagnosed and relapse samples, which were all up-regulated in relapse samples (**Figures 1A–C**). Moreover, 125 GO terms (107 BP and 18 MF) and 4 KEGG pathways were enriched by the 23 DE-NMRGs. For example, nucleoside phosphate metabolic process, nucleotide metabolic process, protein ADP−ribosylation, etc. were the main enriched terms in BP, and pentosyltransferase activity, NAD binding, NAD+ binding, etc, were the main enriched MF terms (**Supplementary Figure S2A**). The 4 enriched KEGG pathways were Nicotinate and nicotinamide metabolism, Nucleotide metabolism, Pyrimidine metabolism, and Purine metabolism (**Supplementary Figure S2B**).

### Three biomarkers were screened out

**Figure 2:**
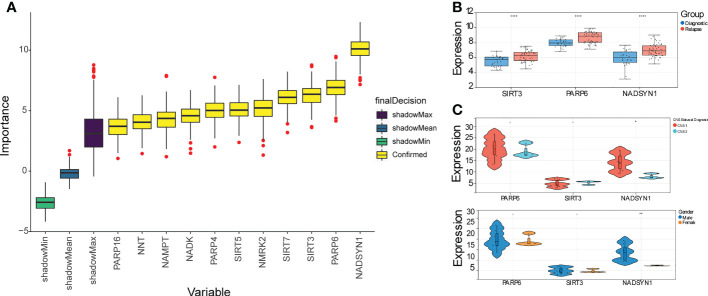
Identification of 3 biomarkers. **(A)** Box plot of importance of 11 DE-NMRGs through Random forest algorithm. **(B)** The expression profile of 3 biomarkers in initially diagnosed and relapse samples. **** represents p < 0.0001. **(C)** The expression levels of 3 biomarkers within different clinical traits (*p* < 0.05). * represents p < 0.05, ** represents p < 0.01, - represents no significant difference.

A total of 11 DE-NMRGs with confirmed importance were screened out by RF, which contained PARP16, NNT, NAMPT, NADK, PARP4, SIRT5, NMRK2, SIRT7, SIRT3, PARP6, and NADSYN1 (**Figure 2A**). Moreover, these 11 DE-NMRGs were input into repeat cross validation, and it can be found that when the accuracy reached the highest point, three genes with the highest importance were selected as biomarkers (NADSYN1, PARP6, SIRT3) (**Supplementary Figure S3A**). The expressions of 3 biomarkers were significantly higher in relapse group (**Figure 2B**). The area under the curve (AUC) of the ROC curve was 0.8031 (95% CI, 0.7024-0.8884), which indicated the diagnostic ability of the 3 biomarkers was great (**Supplementary Figure S3B**), the specificity and sensitivity of AUC for predicting B-ALL were noted to be 78.125 and 67.92453. In addition, the comparison between the expression levels of biomarkers and different clinical traits results demonstrated that the expression of NADSYN1 was significantly different in CNS Status and Gender, which was higher in CNS 1 and Males, compared to CNS 2 and Females respectively (**Figure 2C**).

### TME analysis

**Figure 3:**
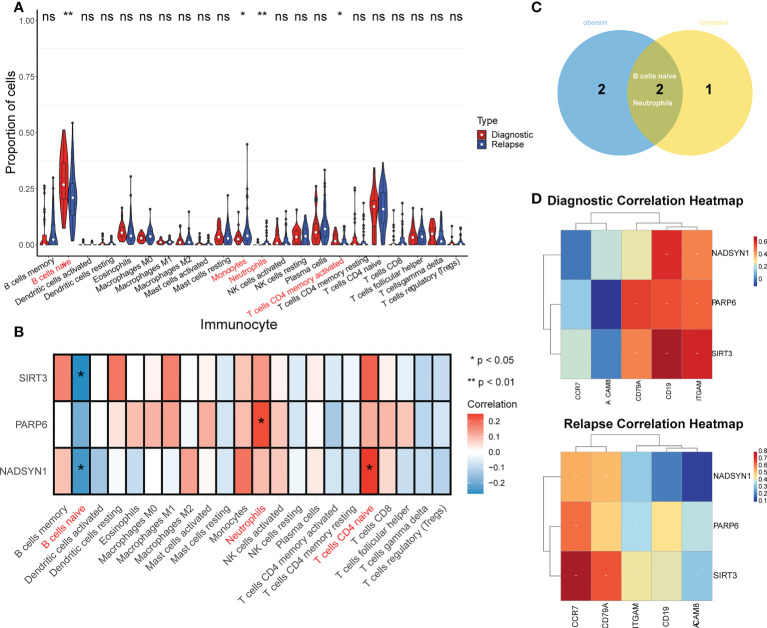
TME analysis in GSE3912. **(A)** Immune infiltration differences between initially diagnosed and relapse groups (*p* < 0.05). * represents p < 0.05, ** represents p < 0.01, ns represents no significance. **(B)** The correlation analysis of immune cells and NADSYN1, PARP6, SIRT3. **(C)** A Venn-diagram of the differentially expressed immune cells (DEIs) and significantly biomarkers-related immune cells to obtain key DEIs. **(D)** The correlation heatmaps of biomarkers and corresponding gene markers of key DEIs in initially diagnosed and relapse samples.

It can be found that the proportion of B cell naive accounted for the largest ratio (**Supplementary Figure S4**), and the proportion differences in B cells naive, Monocytes, Neutrophils, and T cells CD4 memory activated were significantly different between initial diagnosis and relapse groups (**Figure 3A**). Moreover, the correlation analysis result suggested that the 3 biomarkers were significantly correlated with B cells naive, Neutrophils, and T cells CD4 naive. To be more specific, both SIRT3 and NADSYN1 were negatively correlated with B cells naive. In contrast, PARP6 and NADSYN1 were positively correlated with Neutrophils, and T cells CD4 naive respectively (**Figure 3B**).

In addition, the overlap analysis detected 2 key DEIs, which were B cells naive and Neutrophils (**Figure 3C**). In terms of the correlation analysis, the gene markers of B cells were CD19 and CD79A, and the gene markers of Neutrophils were CEACAM8, ITGAM, and CCR7, and NADSYN1 in the initial diagnosis group was positively related to CD19 and ITGAM (26, 27). NADSYN1 in the relapse group was positively correlated with CD79A, ITGAM, and CCR7. In terms of PARP6, in the initial diagnosis group, it was positively correlated with CD79A, CD19, and ITGAM, and it in the relapse groups was positively correlated with all 5 gene markers of the 2 key DEIs. It was noteworthy that the correlation analysis results of SIRT3 were similar to the PARP6, SIRT3 in both groups was positively correlated with CD79A, CD19, and ITGAM, but SIRT3 in the relapse group was also positively correlated with CEACAM8 and CCR7 (**Figure 3D** and **Table 1**).

**Table 1:** The correlation analysis of 3 biomarkers and 2 key differentially expressed immune cells (DEIs).

		NADSYN1				PARP6				SIRT3			
		Diagnostic		Relapse		Diagnostic		Relapse		Diagnostic		Relapse	
		cor	pvalue	cor	pvalue	cor	pvalue	cor	pvalue	cor	pvalue	cor	pvalue
**B cells**	**CD19**	0.604160941	0.000250578	0.202628608	0.145345482	0.552785924	0.001238677	0.459442025	0.000615793	0.742668622	2.96284E-06	0.421472776	0.001672054
	**CD79A**	0.278434608	0.122807739	0.518394582	7.00252E-05	0.576979472	0.000681624	0.48339952	0.000245773	0.465909091	0.007765214	0.661855421	6.8005E-08
**Neutrophils**	**ITGAM**	0.469251216	0.006741144	0.331881955	0.015577313	0.514296188	0.002950867	0.367360103	0.007115302	0.62170088	0.000199657	0.434696716	0.001143773
	**CEACAM8**	0.227785443	0.20989854	0.11652991	0.405997495	-0.113332128	0.536844714	0.377613358	0.005311082	0.023840092	0.896952062	0.321915325	0.018740807
	**CCR7**	0.007882676	0.965846909	0.54320042	2.63654E-05	0.165337733	0.365822526	0.628472363	4.68956E-07	0.238474935	0.188710171	0.818385244	7.23533E-14

### Cluster and pseudotime analysis

After the filtering by Seurat, 76,227 cells, each containing 22,324 genes, were screened out from the 7 pairs of samples in GSE130116 (**Supplementary Figure S5A**), and the top 2000 genes with the greatest variance were selected (**Supplementary Figure S5B**). Additionally, the PCA result showed the overall distribution of the cells was basically consistent, and there was no outlier sample, which could meet the needs of subsequent analyses (**Supplementary Figure S5C**). It can be observed that the p values of the 20 components were all less than 0.05, so the top 10 core cells were selected (**Supplementary Figure S5D**).

**Figure 4:**
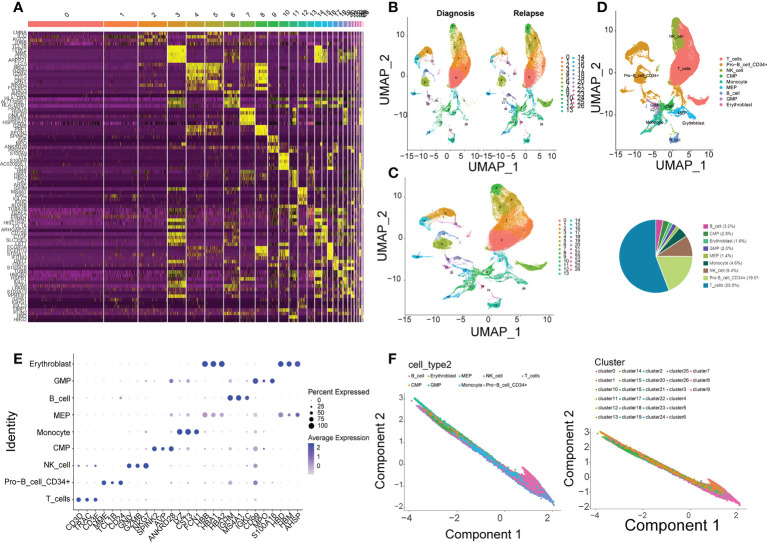
Single-cell analysis in GSE130116. **(A)** 26 clusters of core cells are identified in GSE130116 by UMAP2 algorithm. **(B)** The ratio of 26 clusters in initially diagnosed and relapse samples. **(C)** Heatmap of the expressions of top 5 gene markers in each cluster. **(D)** Functional annotations for 26 clusters by CellMarker. **(E)** Bubble plot of top 3 gene markers in each cell subset. **(F)** Visualization of inferred temporal trajectory of different cell types.

The cluster result of core cells showed that the core cells were clustered into 26 clusters (**Figure 4A**), and the cluster results of samples in initial diagnosis and relapse groups illustrated that most of the clusters showed a similar trend, but there were differences in cluster 19, 24, and 25 (**Figure 4B**). Additionally, the expressions of top 5 gene markers in each cluster were selected for plotting the heat map, and it can be found the expressions of the majority of marker genes were low in clusters (**Figure 4C**).

Furthermore, 9 types of cells were annotated from CellMarker database, which included T cells, Pro-B cell CD34+, NK cell, Monocyte, MEP, GMP, Erythroblast, CMP, and B cell. It can be clearly seen that the percentage of T cells was the most, which accounted for 55.9%, followed by Pro-B cell CD34+ (19.0%) and NK cell (9.4%) (**Figure 4D**). The top 3 gene markers in each of the 9 cell types were displayed in **Figure 4E**, and it can be found that different types of cells had different gene markers. The expression of each biomarker in each cell types were shown in **Supplementary Figure S5E**, they were higher expressed in T cells, Pro-B cell CD34+, GMP, and MEP. The 9 types of cells were projected onto one branch to construct single cell trajectory diagram (**Figure 4F**). In summary, the results of quasi-temporal analysis for the 9 types of cells showed MEP, Pro-B cell CD34+ and CMD were the main cells before the differentiation, and NK-cell, Monocyte, and T cells were the major types after that.

### Interactions among cells

**Figure 5:**
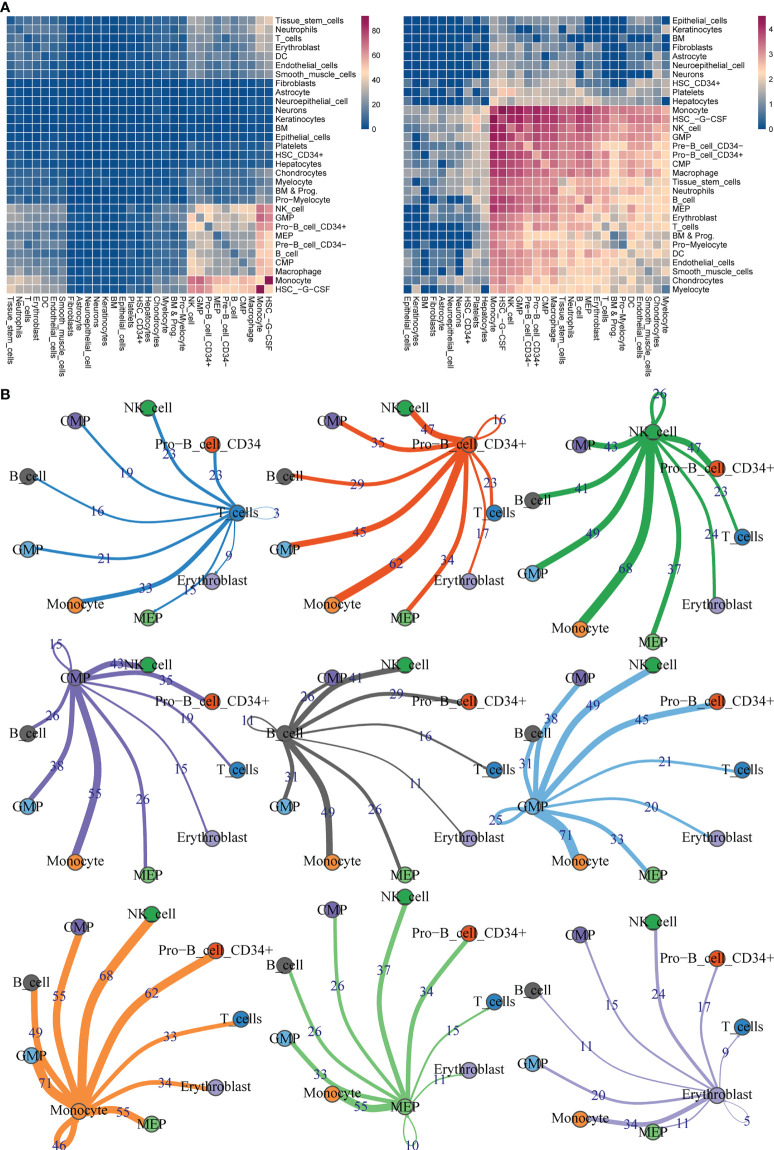
Ligand-receptor interaction predictions between 9 cell types. **(A)** Heatmaps of ligand–receptor interactions by CellphoneDB analysis. **(B)** Visualization of interaction pairs with *p* value <= 0.05 and log2 mean (Molecule 1, Molecule 2) >= 0.1.

The number of ligand-receptor interactions and polymers was displayed in heatmaps (**Figure 5A**). After filtration and screening, the interaction pairs of the 9 types of cells were obtained and plotted. For instance, Monocytes strongly interacted with GMP, and there were 71 interacted ligands, receptors, and polymers between them (**Figure 5B**).

### Confirmation of key cell clusters and GSEA

**Figure 6:**
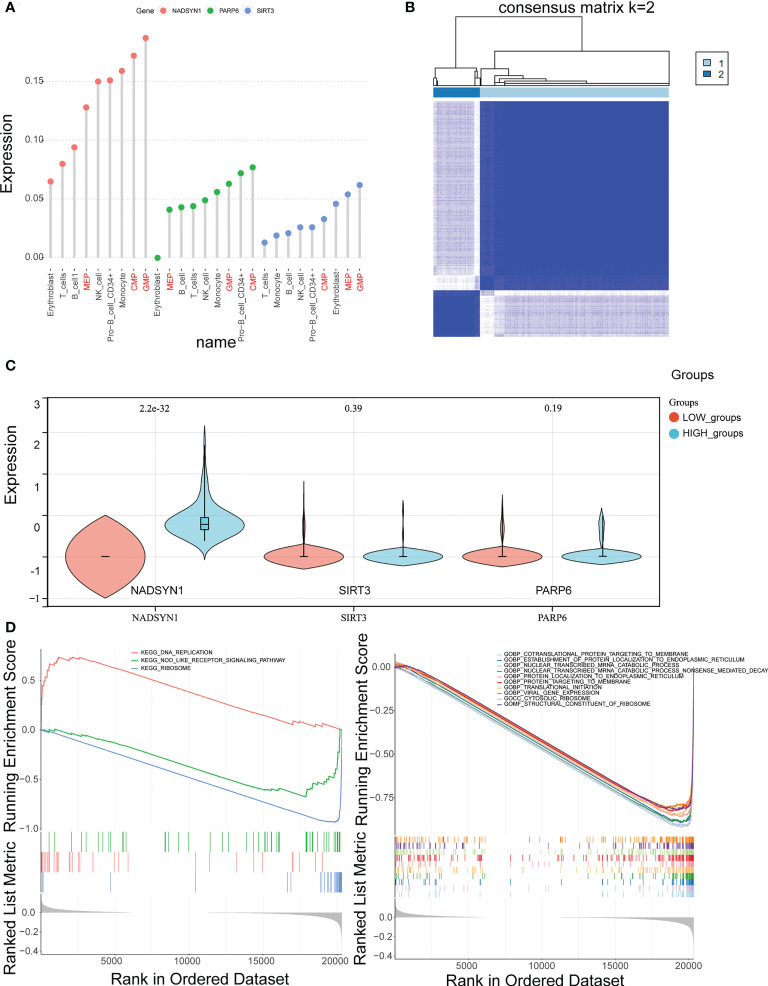
Identification of key cell clusters. **(A)** The distribution of the biomarkers expressions in different cell types to identify key cell clusters. **(B)** Correlation between the two clusters (k = 2). **(C)** The expression levels of biomarkers in the high- and low- expression groups. **(D, E)**. Gene Set Enrichment Analysis (GSEA) in the high- and low-expression groups.

Initially, the rank of the biomarkers’ expressions was presented in **Figure 6A** and **Supplementary Figure S6A**, considering the expression levels of the three biomarkers, the expression levels of them in CMP, GMP and MEP were the highest. Therefore, CMP, GMP, and MEP cell clusters were selected as the key cell clusters with a total of 5,563 cells, and the Consensus Clustering revealed the K = 2 (**Figure 6B**, **Supplementary Figure S6B**). Subsequently, the biomarkers expressions of the 2 clusters in the consensus clustering were plotted, which indicated the expression of NADSYN1 was significantly higher in cluster 2, so cluster 1 and cluster 2 were deemed as low biomarker expression group and high biomarker expression group respectively (**Figure 6C**). Moreover, the low biomarker expression group represented the major ratio in both initial diagnosis and relapse groups (t = -4.086, p = 0.005) (**Supplementary Figure S6C**).

Furthermore, the GSEA results illustrated that 55 GO terms and 3 KEGG pathways were enriched. The top 10 enriched GO terms were shown, which mainly included Cotranslation protein targeting to membrane, Establishment of protein localization to endoplasmic reticulum, Nuclear transcribed mRNA catabolic process, etc. (**Figure 6D**). The 3 KEGG pathways were DNA replication, Nod like receptor signaling pathway, and Ribosome (**Figure 6E**).

### CREB3L2(+) might be the potential TF affecting the expression of biomarkers

**Figure 7:**
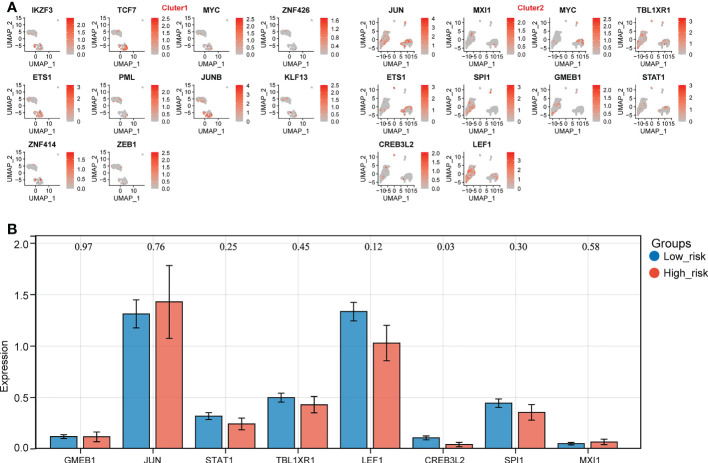
Identification of key transcription factors (TFs) in high- and low- biomarker expression groups. **(A)** The distribution of top 10 TFs in different clusters. **(B)** The expression prfile of key TFs in high- and low- expression groups (p < 0.05).

The SCENIC results were shown in scatter plots and bar charts, and it can be found that MYC(+) and ETS1(+) were the common TFs of the top 10 TFs in both expression groups (**Figure 7A**, **Supplementary Figures S7A, B**). Consequently, the expressions of remained 8 TFs were compared between the 2 expression groups, which indicated that CREB3L2(+) was the only TF that was significantly different between the expression groups, so it was considered as the TF that could affect the expression of biomarkers (**Figure 7B**).

As a result, the potential regulation of the key TF could be stated as follows: in normal cases, CREB3L2(+) could repress the expression of NADSYN1. On the other hand, in the relapse cases, due to the unknown factors, the expression of CREB3L2(+) declined, and its repression effect on NADSYN1 drops as well, which would cause the rise of NADSYN1 expression. The increased NADSYN1 expression could lead to the decrease of CREB3L2(+) expression through the enhancement of negative feedback.

### qPCR validation of biomarkers expressions

**Figure 8:**
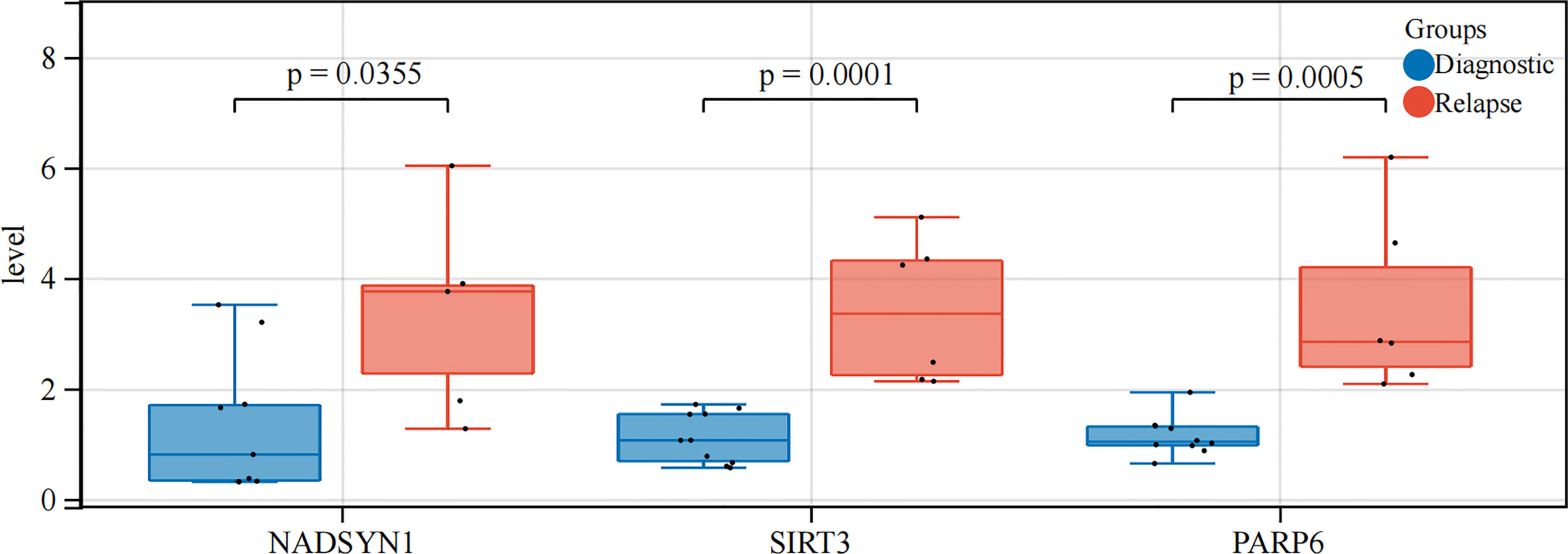
qRT-PCR analysis. The expression levels of NADSYN1, PARP6, SIRT3.

In order to further verify the expression levels of the 3 biomarkers (NADSYN1, PARP6, SIRT3), qPCR was performed between 10 initial diagnoses and 6 relapse B-ALL samples. The expression levels of all the 3 biomarkers were significantly higher in relapse samples (*P* < 0.05), which were consistent with the previous bioinformatics differential analysis in GEO (**Table 2** and **Figure 8**).

**Table 2:** Expression analysis between initial diagnosis and relapse samples.

	IG	RG	t, df	p
**NADSYN1**	1.3527 ± 1.2641	3.3436 ± 1.9010	t=2.369 df=12	0.0355
**SIRT3**	1.1102 ± 0.4585	3.4049 ± 1.3023	t=5.163 df=14	0.0001
**PARP6**	1.1359 ± 0.3512	3.4705 ± 1.6079	t=4.515 df=14	0.0005

## Discussion

B-ALL is the most common hematological malignancy in children, and relapse is an important adverse factor affecting the survival of patients. About 20% of treated B-ALL patients will relapse and 10% of patients with diagnosed with ALL remain incurable (3). It has been reported that NMRGs in whole blood transcriptome data are biomarkers to predict clinical outcomes in muscular atrophy patients (14), and S Takao et al. have found that depletion of NAD + by KPT-9274 inhibitors may be a promising alternative to treat B-ALL patients (13). Moreover, Sarah K Tasianet al. have also expounded on the biological role of genomic and epigenomic analysis in the initial diagnosis and recurrent ALL (4). However, the mechanism of NMRGs in B-ALL recurrence is still unclear. Therefore, exploring NMRGs has biological importance in the initial diagnosis and recurrence of B-ALL.

Three key biomarkers: NADSYN1, SIRT3, and PARP6 were identified, and the ROC curve suggested they had a good ability to diagnose initial diagnosis and relapse samples. NAD synthetase (EC 6.3.5.1) catalyzes the final step in the biosynthesis of NAD from nicotinic acid adenine dinucleotide (NaAD).SIRT3 encodes a member of the sirtuin family of class III histone deacetylases, homologs to the yeast Sir2 protein. SIRT3 has far-reaching effects on nuclear gene expression, cancer, cardiovascular disease, neuroprotection, aging, and metabolic control.PARP6 enables protein ADP-ribosylase activity. Involved in protein auto-ADP-ribosylation and protein mono-ADP-ribosylation. Although previous studies have found that NADSYN1 is closely associated with congenital diseases such as vertebral malformations, multiple organ defects, and multiple sclerosis (28–30). While SIRT3 was found to be a novel regulator of cardiovascular disease (31), and PARP6 has suggested inhibiting the development of hepatocellular carcinoma and intestinal cancer (32, 33). Eguchi-Ishimae et al. found that a unique allele (quinone oxidoreductase, NQO1) of NAD (P)H was associated with pediatric acute lymphoblastic leukemia and the mixed lineage leukemia(MLL) fusion gene in Japanese (34), and Slah Ouerhani also found that in ALL patients. Besides, the NQO1 609CT genotype was overrepresented in ALL patients compared with the reference group of the NQO1 609CC genotype, and it is firmly believed that the occurrence of ALL is related to the metabolic and environmental exposure of carcinogens (35).

However, the relationship between NADSYN1, SIRT3, and PARP6 and the recurrence mechanism of B-ALL has not been reported. The authors speculated that it may be after a relapse of B-ALL, the up-regulation of three metabolic genes of NADSYN1, PARP6 and SIRT3, was promoted in response to certain tumor factors. Leading to an increasing NAD metabolism. This metabolic change can induce a higher pro-inflammatory senescence-associated secretory phenotype(SASP), which accelerates cancer progression in surrounding cells (12), and leads to the malignant proliferation of naive B lymphocytes. In addition, NADSYN1 also found significant differences in clinical traits in B-ALL such as CNS_Status and Gender, and with significantly increased expression in CNS1 and men.Sirvent et al. found in pediatric ALL that CNS-3 status remains an independent poor outcome factor in a randomized EORTC 58951 trial study including 1930 ALL patients (36). Joni Van der Meulen et al. have proved that the H3K27me3 demethylase UTX is a sex-specific tumor suppressor in pediatric T-ALL, while the UTX mutation is only present in male T-ALL patients (37). Nevertheless, there are few researches in this field, and this study may be the first discovery, but a large number of studies concentrated on this topic are still needed.

Next, through CIBERSORT algorithm, two immune cell types (B cells naive and Neutrophils) were identified that differentially expressed in childhood ALL between initial diagnosis and relapse and that were associated with biomarkers. NADSYN1 and SIRT3 were negatively correlated with B cells naive, and PARP6 was positively correlated with Neutrophils. However, Kavita Bhalla et al. found that SIRT3 (sirtuin 3) is a metabolic target related to the defect of ataxia-telangiectasia mutated (ATM) gene in diffuse large B cell lymphoma. The increased activity of SIRT3 will lead to the disorder of mitochondrial structure and reduced tricarboxylic acid flux (38). SIRT3 is the major deacetylase in the mitochondrial matrix, by deacetylation and activating isocitrate dehydrogenase 2 (IDH2) and superoxide dismutase 2 (SOD2). Wei Yu et al. believe that SIRT3 is a tumor suppressor in B-cell malignancies, activates the SIRT3 pathway, promotes aerobic metabolism and controls reactive oxygen species (ROS), and suppresses the hypoxia-inducing factor-1-independent mechanism that may be a new therapeutic method for the treatment of B-cell malignancies (39). However, there is no reported association of NADSYN1, SIRT3, and B cells naive, or in the association of PARP6 or Neutrophils in B-ALL patients. The authors speculated that in patients with B-ALL relapse, SIRT3 and NADSYN1 were up-regulated in response to certain tumor factors. On the one hand, by increasing NAD metabolism to promote the proliferation of B cells naïve population, On the other hand, with the gradual proliferation of the B cell naïve population, it released certain negative feedback regulators, this resulted in the inhibition of SIRT3 and NADSYN1 expression, and the negative feedback effect was stronger than the effect of some tumor factors. Meanwhile, in patients with B-ALL relapse, neutrophil proliferation is suppressed due to malignant expansion of B lymphocytes. And in order to achieve the need of body compensation, under the action of some certain regulatory factors, promoting PARP6 up-regulation, and promote proliferation of neutrophil by enhancing NAD metabolism. Hiroto Inaba et al. mention that cellular immunotherapy for ALL is the future development direction (40), and the research also found that ALL can be treated by consuming NADH (13). Therefore, combined with our findings between biomarkers and immune cells, there is a correlation between NMRGs and immune cells in B-ALL.

Single-cell analysis was then performed in the GSE130116 dataset, annotated to nine types of cells: T_cells, Pro-B_cell_CD34 +, NK_cell, Monocyte, MEP, GMP, Erythroblast, CMP, B_cell, and found the interaction ligand-receptor, polysome between cell subtypes. No reports have been reported interrelationship between Monocyte and GMP in B-ALL patients. However, K Akashi et al. purified common cocloned myeloid progenitor cells by using cell surface labeling and flow cytometry, and determined a differentiated and developmental relationship between Monocyte and GMP (41). In the future research work, we will further focus on the role of the interrelationship between Monocytes and GMP in the mechanism of B-ALL recurrence.

This study found high biomarker expressions in the CMP, GMP, and MEP cell clusters which were identified as the key cell clusters. Finally, we clustered CMP, GMP, and MEP cell clusters according to the biomarkers into high and low expression groups. GSEA enrichment analysis yielded 55 GO enrichment, mainly including co-translational proteins targeting the membrane, protein localization to the ER, mRNA catabolism process, and three KEGG pathways, DNA replication, Nod-like receptor signaling, and ribosome, respectively. While NAMPT produces NAD, activated PARPs consume the majority of NAD to support their DNA repair activity in response to DNA damaging insults (42). In a human activated T cell and T cell ALL model, PARP inhibitors were found to antagonize NAD depletion and increase NAD levels, thus antagonizing NAMPTi-induced NAD depletion and its downstream effects (43). Similar studies where the cytotoxic effect of FK866 on hematological malignant cells was considered as loss of PARP1 reversed ROS accumulation, mitochondrial depolarization, and loss of ATP (44), while we confirmed a significant enrichment of NMRGs on DNA replication pathways in B-ALL. Yang Hu et al. used RNA-sequencing technology to identify nod-like signaling and leukemia-surface receptor signaling pathways by KEGG annotation, and our study also confirmed that key biomarkers are closely related to Nod-like receptor signaling (9).

Our study also found that the transcription factor CREB3L2 (+) was significantly reduced in the high expression group, and it possibly is the TF affecting the biomarkers in the high expression group. Therefore, we speculated that in recurrent cases, CREB3L2 (+) expression is decreased due to unknown factors, and its inhibitory effect on NADSYN1 is also decreased, which leads to the increased expression of NADSYN1. Further, the increased NADSYN1 expression can lead to decreased CREB3L2 (+) expression through enhanced negative feedback. Surprisingly, no previous studies mention similar results, and we proposed this possible regulation for the first time.

Nevertheless, there is still a limitation in this study. The sample size of RT-qPCR clinical validation is too small, and the analysis results may be biased.

## Data availability statement

Publicly available datasets were analyzed in this study. GSE3912 and GSE130116 datasets were acquired from GEO database (https://www.ncbi.nlm.nih.gov/geo/). NAD+ metabolization-related genes (NMRGs) were selected from Kyoto Encyclopedia of Genes and Genomes (KEGG, http://www.kegg.jp/) (Pathway: hsa00760) and the Reactome (http://reactome.org/, R-HSA-196807) databases. Moreover, 9 pediatric B-ALL samples were acquired from the TARGET (https://ocg.cancer.gov/programs/target) database.

## Ethics statement

The studies involving human participants were reviewed and approved by The Seventh Affiliated Hospital, Sun Yat-Sen University. Written informed consent to participate in this study was provided by the participants’ legal guardian/next of kin.

## Author contributions

CL and J-QX provided equal contributions to conducting the statistical analysis, research design, and drafting the article. CC and G-CZ performed data management and bioinformatics analysis. CL, J-QX, G-CZ, HC, H-MX, MY and CC edited and revised the article. All authors read and approved the final version of the manuscript. CL and J-QX provided equal contributions to the principal investigator, conducted statistical analysis and drafted the article.

## Funding

We thank Sanming Project of Medicine in Shenzhen(No.SZSM202011004), Shenzhen Science and Technology Innovation Commission (JCYJ20210324123004011, JCYJ20180307150419435) and Shenzhen Healthcare Research Project (Grant No. SZLY2018001) for supporting the manuscript preparation and publication.

## Acknowledgments

Thanks to all authors for their contributions to this manuscript.

## Conflict of interest

The authors declare that the research was conducted in the absence of any commercial or financial relationships that could be construed as a potential conflict of interest.

## Publisher’s note

All claims expressed in this article are solely those of the authors and do not necessarily represent those of their affiliated organizations, or those of the publisher, the editors and the reviewers. Any product that may be evaluated in this article, or claim that may be made by its manufacturer, is not guaranteed or endorsed by the publisher.

## Supplementary material

Supplementary Figure 1 The workflow chart of this study.

Supplementary Figure 2 Enrichment analysis.

Supplementary Figure 3 The cross-validation method to screen out 3 biomarkers and ROC curve.

Supplementary Figure 4 The proportions of 22 types of immune cells in GSE3912.

Supplementary Figure 5 Quality control of single-cell RNA-seq (scRNA-seq) data in GSE130116.

Supplementary Figure 6 Consensus Clustering.

Supplementary Figure 7 The regulation specificity scores of top 10 TFs in different expression groups.

Supplementary Table 1 Clinical and Demographic characteristics of B-ALL patients from GSE3912 dataset, GSE130116 dataset, and TARGET database.

Supplementary Table 2 Clinical characteristics of B-ALL patients from our study.

Supplementary Table 3 Primers for qPCR used in the study.
