# Supplementary figures and images for: Integrating RNA-seq and scRNA-seq to explore the biological significance of NAD + metabolism-related genes in the initial diagnosis and relapse of childhood B-cell acute lymphoblastic leukemia
Source: Front Immunol. 2022 Nov 11;13:1043111. doi: 10.3389/fimmu.2022.1043111 (PMC9691973; doi:10.3389/fimmu.2022.1043111)

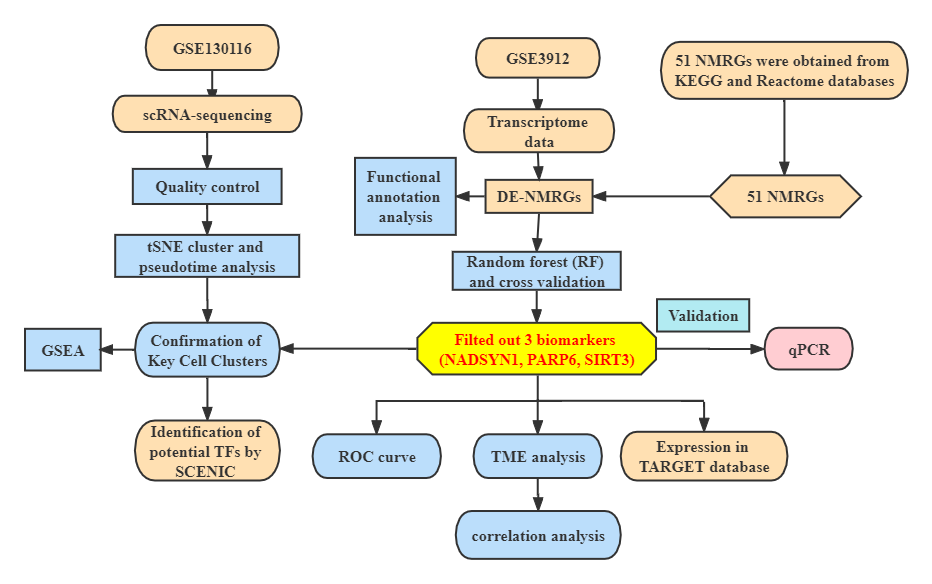

Supplement: Supplementary Figure 1 — The workflow chart of this study. [file Image_1.tif]

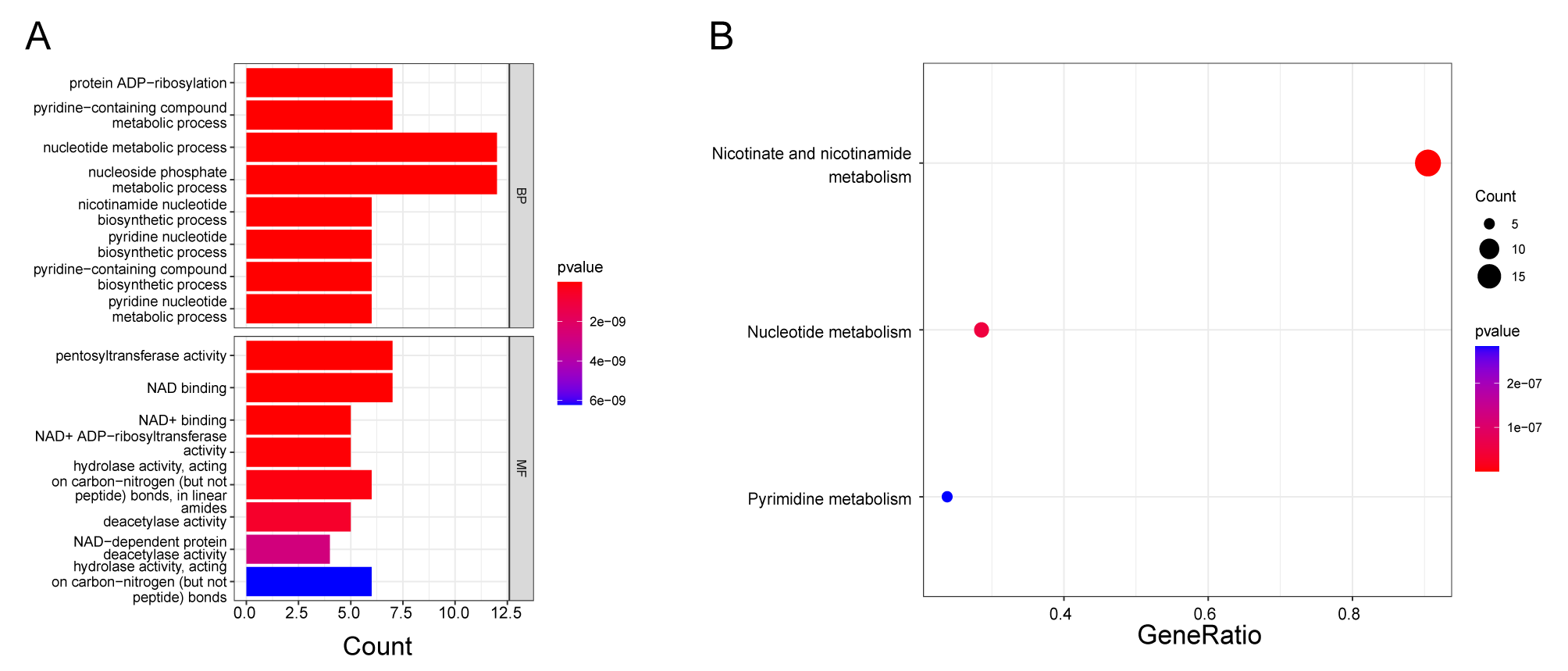

Supplement: Supplementary Figure 2 — Enrichment analysis. [file Image_2.tif]

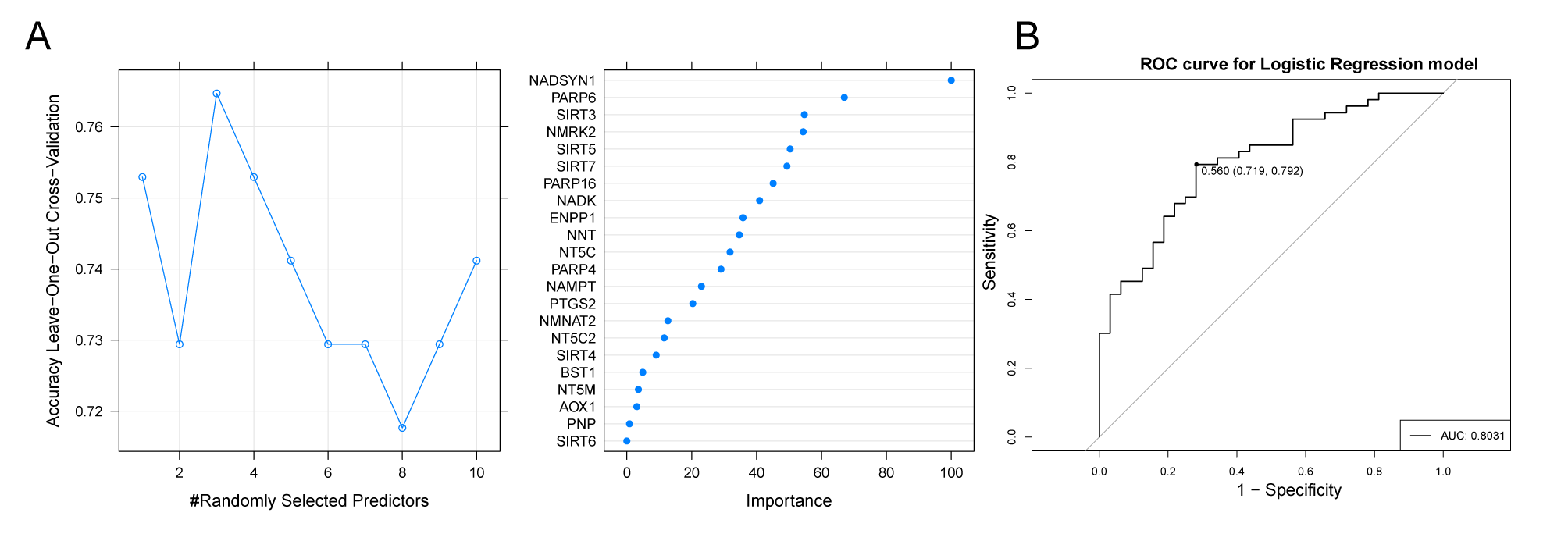

Supplement: Supplementary Figure 3 — The cross-validation method to screen out 3 biomarkers and ROC curve. [file Image_3.tif]

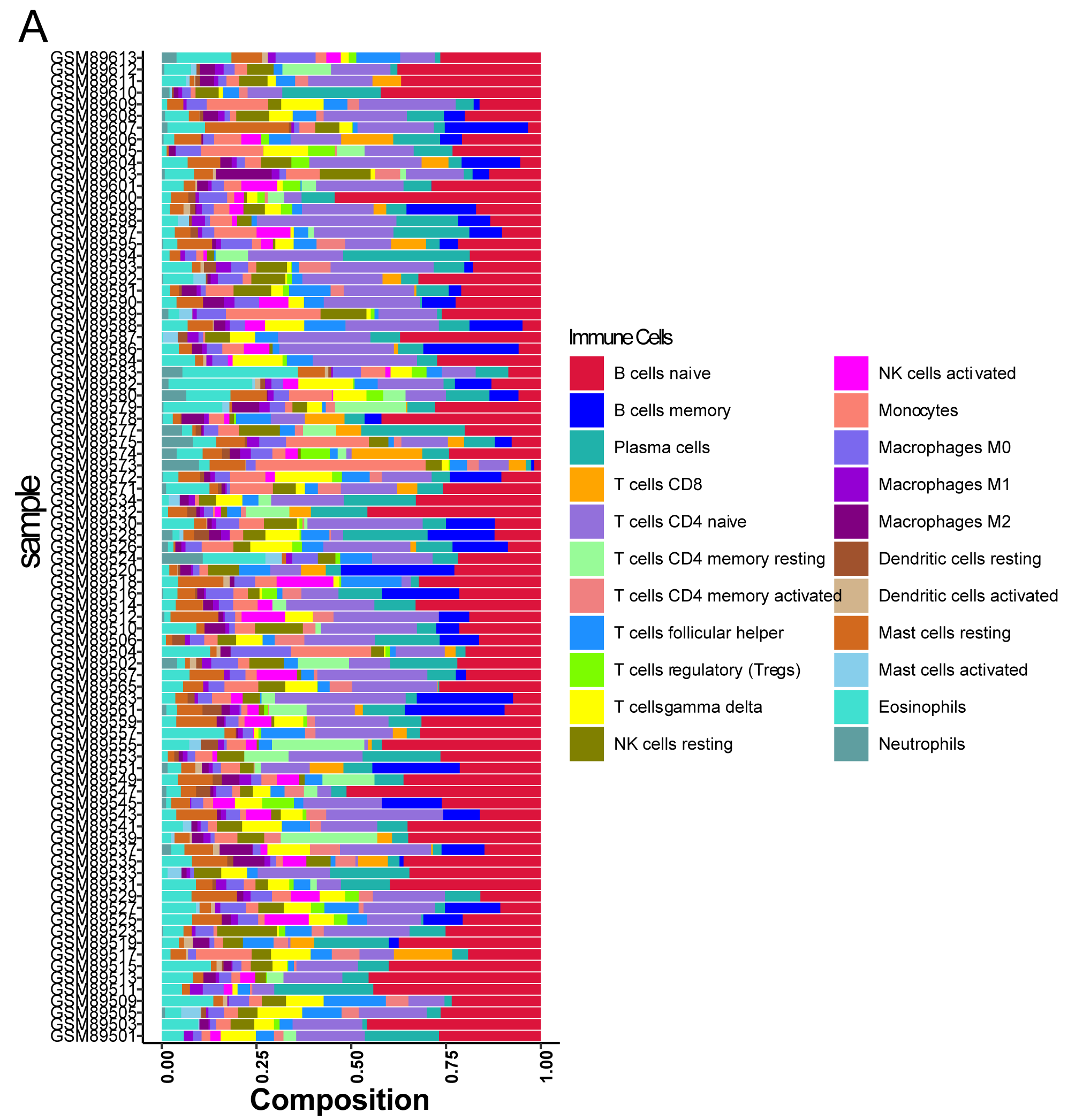

Supplement: Supplementary Figure 4 — The proportions of 22 types of immune cells in GSE3912. [file Image_4.tif]

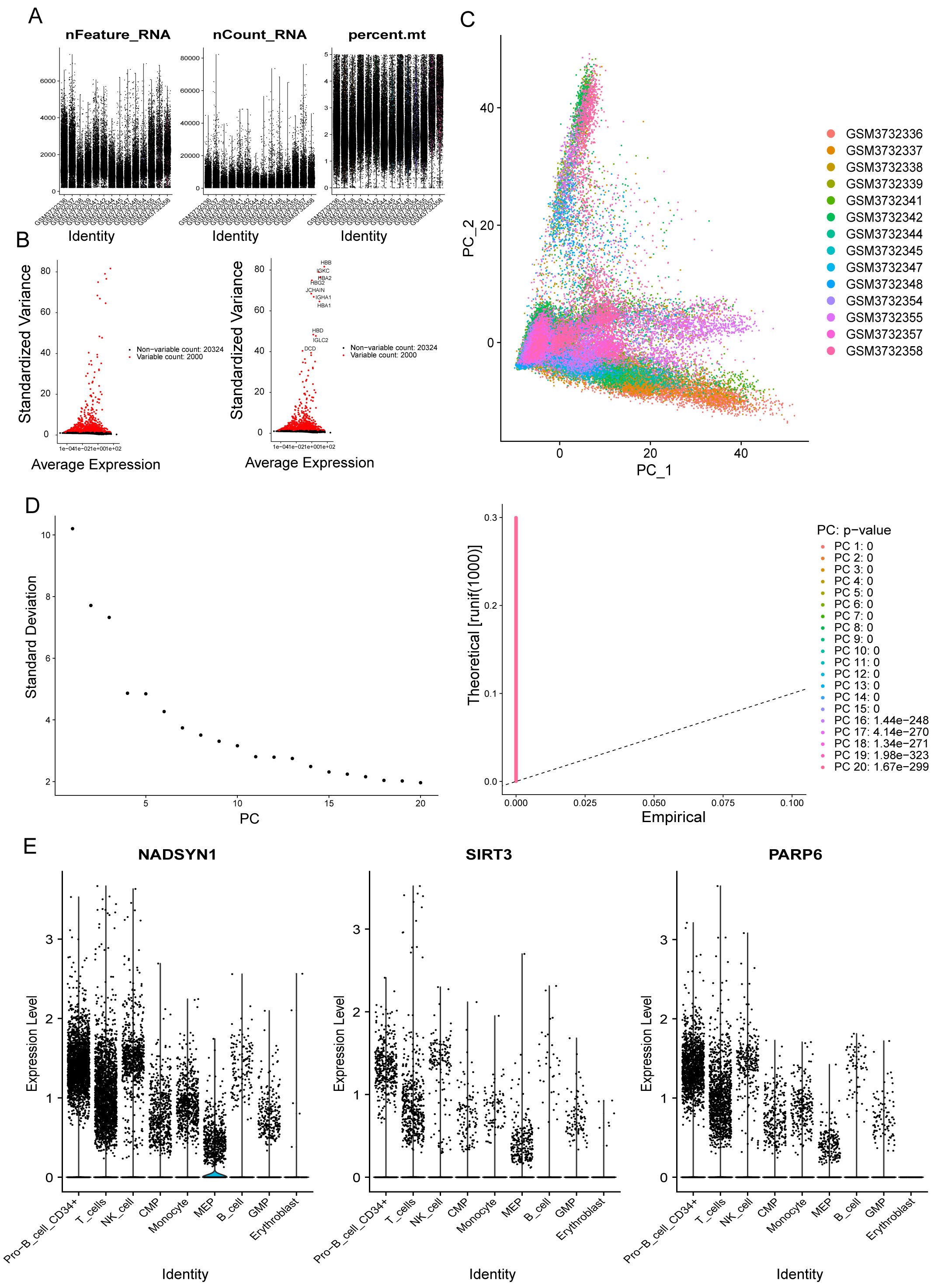

Supplement: Supplementary Figure 5 — Quality control of single-cell RNA-seq (scRNA-seq) data in GSE130116. [file Image_5.tif]

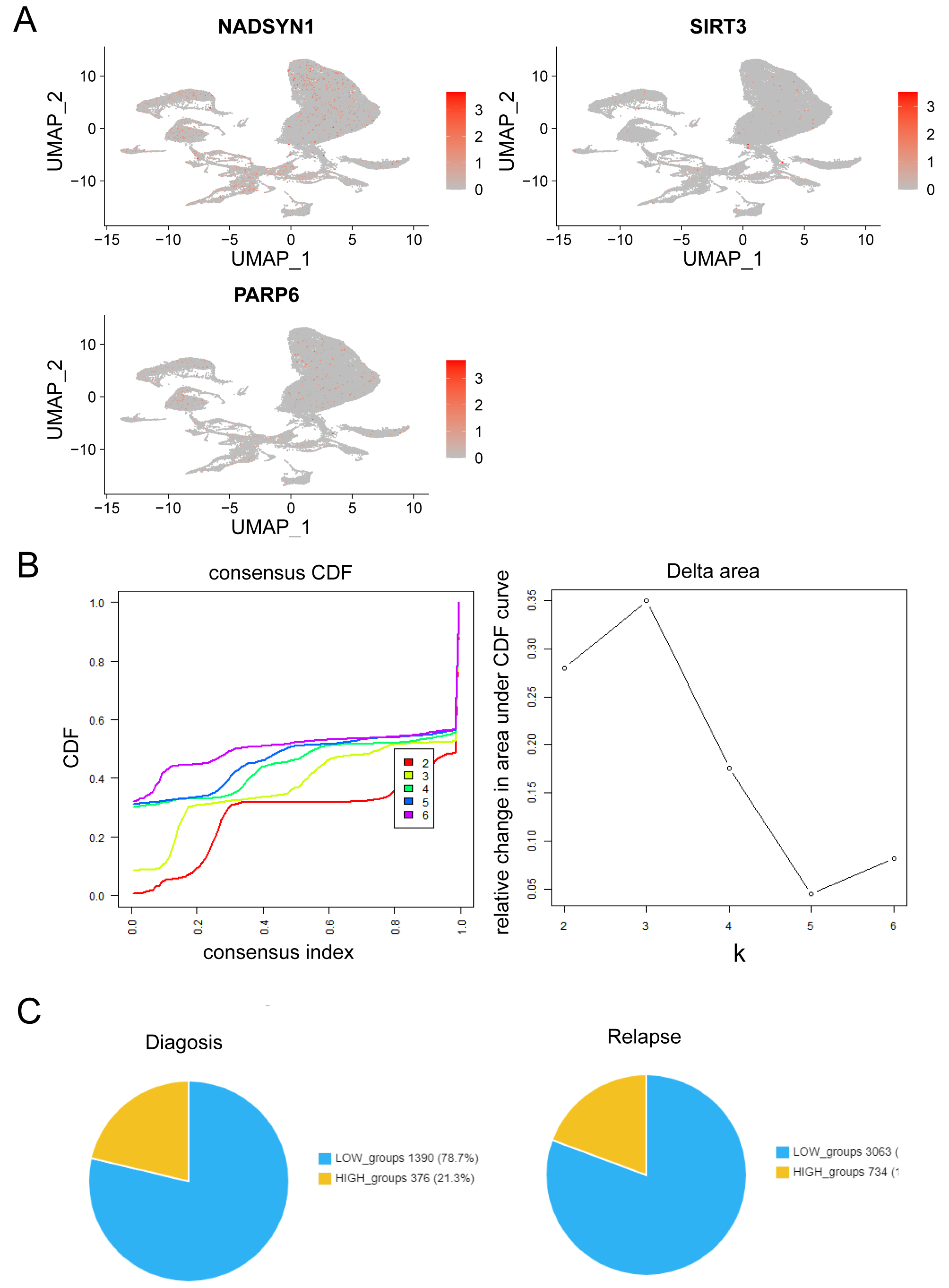

Supplement: Supplementary Figure 6 — Consensus Clustering. [file Image_6.tif]

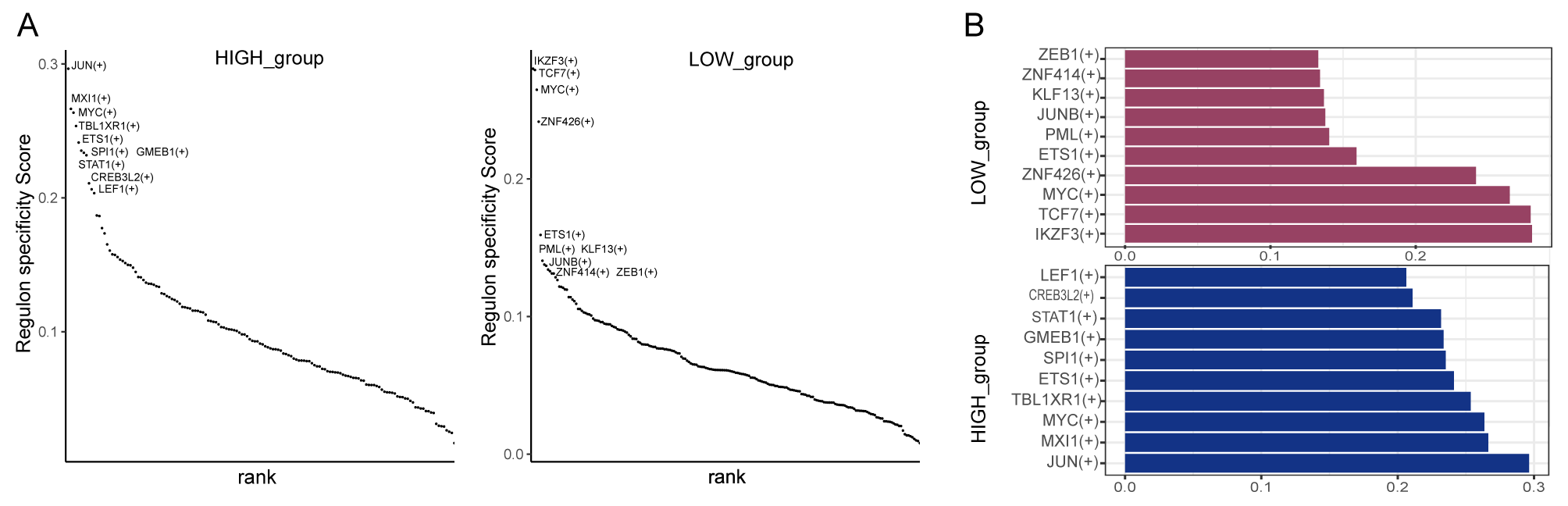

Supplement: Supplementary Figure 7 — The regulation specificity scores of top 10 TFs in different expression groups. [file Image_7.tif]
