# Supplementary material for: Integrating RNA-seq and scRNA-seq to explore the biological significance of NAD + metabolism-related genes in the initial diagnosis and relapse of childhood B-cell acute lymphoblastic leukemia
Source: Front Immunol. 2022 Nov 11;13:1043111. doi: 10.3389/fimmu.2022.1043111 (PMC9691973; doi:10.3389/fimmu.2022.1043111)
Supplement: Supplementary file 11 [file DataSheet_1.doc]

1.Raw data

<https://www.jianguoyun.com/p/DUS6wigQ3qn4ChiTnNcEIAA>

2.Raw data results

<https://www.jianguoyun.com/p/DaM84rAQ3qn4Chjdm9cEIAA>

3.PCR results

<https://www.jianguoyun.com/p/DXK6Br8Q3qn4Chjam9cEIAA>

4.R

<https://www.jianguoyun.com/p/DVU76UQQ3qn4ChjXm9cEIAA>
